# Comprehensive Biological Evaluation of Biomaterials Used in Spinal and Orthopedic Surgery

**DOI:** 10.3390/ma13214769

**Published:** 2020-10-26

**Authors:** Piotr Komorowski, Małgorzata Siatkowska, Marta Kamińska, Witold Jakubowski, Marta Walczyńska, Magdalena Walkowiak-Przybyło, Witold Szymański, Katarzyna Piersa, Patryk Wielowski, Paulina Sokołowska, Kamila Białkowska, Krzysztof Makowski, Marcin Elgalal, Agnieszka Kierzkowska, Lechosław Ciupik, Bogdan Walkowiak

**Affiliations:** 1 Molecular and Nanostructural Biophysics Laboratory, “Bionanopark” Ltd., Dubois 114/116, 93-465 Lodz, Poland; m.siatkowska@bionanopark.pl (M.S.); k.dzialoszynska@bionanopark.pl (K.P.); wielowskipatryk@gmail.com (P.W.); p.sokolowska@bionanopark.pl (P.S.); k.bialkowska@bionanopark.pl (K.B.); m.elgalal@bionanopark.pl (M.E.); bogdan.walkowiak@p.lodz.pl (B.W.); 2 Division of Biophysics, Institute of Materials Science, Lodz University of Technology, Stefanowskiego 1/15, 90-924 Lodz, Poland; marta.kaminska@p.lodz.pl (M.K.); witold.jakubowski@p.lodz.pl (W.J.); marta.walczynska@p.lodz.pl (M.W.); magdalena.walkowiak-przybylo@p.lodz.pl (M.W.-P.); witold.szymanski@p.lodz.pl (W.S.); 3 Department of Medical Imaging Technique, Medical University of Lodz, Lindleya 8, 90-419 Lodz, Poland; 4 Department of Pharmacology and Toxicology, Medical University of Lodz, Zeligowskiego St. 7/9, 90-752 Lodz, Poland; 5 Department of General Biophysics, University of Lodz, Pomorska 141/143, 90, 90-236 Lodz, Poland; 6 Industrial Biotechnology Laboratory, “Bionanopark” Ltd., Dubois 114/116, 93-465 Lodz, Poland; k.makowski@bionanopark.pl; 7 Department of Diagnostic Imaging, Radiation and Isotope Therapy, Medical University of Lodz, Pomorska 251, 92-215 Lodz, Poland; 8 “LfC” Ltd., Kozuchowska 41, 65-364 Zielona Gora, Poland; a.kierzkowska@lfc.com.pl (A.K.); l.ciupik@lfc.com.pl (L.C.)

**Keywords:** materiomics, biocompatibility, transcriptome, proteome, spine surgery

## Abstract

Biological acceptance is one of the most important aspects of a biomaterial and forms the basis for its clinical use. The aim of this study was a comprehensive biological evaluation (cytotoxicity test, bacterial colonization test, blood platelets adhesion test and transcriptome and proteome analysis of Saos-2 cells after contact with surface of the biomaterial) of biomaterials used in spinal and orthopedic surgery, namely, Ti6Al4V ELI (Extra Low Interstitials), its modified version obtained as a result of melting by electron beam technology (Ti6Al4V ELI-EBT), polyether ether ketone (PEEK) and polished medical steel American Iron and Steel Institute (AISI) 316L (the reference material). Biological tests were carried out using the osteoblasts-like cells (Saos-2, ATCC HTB-85) and bacteria *Escherichia coli* (DH5α). Results showed lack of cytotoxicity of all materials and the surfaces of both Ti6Al4V ELI and PEEK exhibit a significantly higher resistance to colonization with *E. coli* cells, while the more porous surface of the same titanium alloy produced by electron beam technology (EBT) is more susceptible to microbial colonization than the control surface of polished medical steel. None of the tested materials showed high toxicity in relation to *E. coli* cells. Susceptibility to platelet adhesion was very high for polished medical steel AISI 316L, whilst much lower for the other biomaterials and can be ranked from the lowest to the highest as follows: PEEK < Ti6Al4V ELI < Ti6Al4V ELI-EBT. The number of expressed genes in Saos-2 cells exposed to contact with the examined biomaterials reached 9463 genes in total (ranging from 8455 genes expressed in cells exposed to ELI to 9160 genes in cells exposed to PEEK). Whereas the number of differentially expressed proteins detected on two-dimensional electrophoresis gels in Saos-2 cells after contact with the examined biomaterials was 141 for PEEK, 223 for Ti6Al4V ELI and 133 for Ti6Al4V ELI-EBT. Finally, 14 proteins with altered expression were identified by mass spectrometry. In conclusion, none of the tested biomaterials showed unsatisfactory levels of cytotoxicity. The gene and protein expression analysis, that represents a completely new approach towards characterization of these biomaterials, showed that the polymer PEEK causes much more intense changes in gene and protein expression and thus influences cell metabolism.

## 1. Introduction

It is well known that bone tissue is constantly subjected to the dynamic processes of remodeling. This process is carried out by osteoblasts, which produce and secrete matrix proteins and transport minerals into the bone matrix, as well as osteoclasts that break down bone tissue. Osteolysis is defined as the physiological process of active resorption of bone tissue by osteoclasts during bone remodeling; however, it is also understood as the pathological destruction or resorption of bone tissue that is in direct contact with an implant and is recognized as one of the most serious complications following bone surgery. It is widely believed that post-surgery osteolysis is caused by the presence of debris resulting from the wear of implants [1,2,3]. On the other hand, besides the presence of debris, many other factors such as age, mechanical stress or synovial fluid pressure may also cause osteolysis [4]. It cannot be ruled out that direct contact of an implant surface with bone tissue has an impact on the process of bone remodeling and osteointegration. This effect may possibly be manifested by changes in gene expression caused by exposure and direct interaction of osteoblasts with an implant surface. Moreover, it is becoming increasingly obvious that a holistic approach must be applied in order to understand the varied processes that determine the successful use of a particular implant. Such a comprehensive evaluation allows for multilevel and multiscale analysis of cellular response to contact with a biomaterial surface that has a predetermined, well-defined structure. This all-inclusive approach is the essence of materiomics [5,6,7,8] and gene expression analysis is a crucial component of this process [9]. It would seem that most, commonly used biomaterials have already been thoroughly and sufficiently characterized for the purpose of using these materials in bone and spine surgery. However, a detailed analysis of papers that have been published to date, indicates the difficulties in comparing this data, due to the variety of research techniques and experimental conditions used in these studies. In addition, there is no available information concerning tissue response observed at the molecular level. For this reason, we decided to perform a comprehensive biological evaluation of two representative and commonly used biomaterials—Ti6Al4V Extra Low Interstitials (ELI) alloy (metal) and polyether ether ketone (PEEK) (polymer). Bearing in the mind the possible effect of biomaterial surface structure on cellular response, a decision was made to additionally use titanium alloy samples produced using electron beam technology (Ti6Al4V ELI-EBT). Medical steel was included in the study as the internal laboratory standard.

Since bone is a highly vascular tissue and blood platelets are a potent source of growth factors, it was decided that both osteoblasts and blood platelets would be used for this evaluation. An additional factor that determines the usefulness of biomaterials is their resistance to formation of microbial biofilm on their surfaces, thus reducing the risk of microbial infections. Consequently, bacteria cells were also included in these studies. The above-mentioned biomaterials were subjected to both structural and biological assessment. Scanning electron microscopy (SEM) with energy-dispersive X-ray spectroscopy (EDS) were used for biomaterial characterization. Citrated human blood was used for the blood platelet adhesion test; osteoblasts were used for determination of cytotoxicity, as well as for the study of transcriptome and proteome profiles, whereas bacteria were used for assessment of microbial colonization on the surfaces of these materials.

## 2. Materials and Methods

### 2.1. Biological Materials

For the purpose of this study human blood was obtained from healthy volunteers who had not used any antiplatelet medication for at least 2 weeks prior to the experiment. The study was conducted with the consent of the Local Ethics Committee at the Medical University of Lodz, Poland (RNN/46/06/KB 21 February 2006). Human osteosarcoma cell line Saos-2 (ATCC HTB-85) was obtained from American Type Culture Collection (ATCC, Manassas, VA, USA), whereas bacteria *Escherichia coli* strain DH5α (18265017) was acquired from Thermo Fisher Scientific Inc. (Waltham, MA, USA).

### 2.2. Chemicals and Disposables

Bactopeptone, bis-benzimide, glutaraldehyde, penicillin, propidium iodide, sodium citrate, streptomycin, trypsin, and yeast extract were acquired from Sigma Aldrich (Saint Luis, MO, USA); anti CD61-PerCP, anti CD62-PE, and PAC-1-FITC (Becton-Dickinson, Franklin Lakes, NJ, USA); Hoechst 33342 from Santa Cruz Biotechnology (Dallas, TX, USA); McCoy’s 5A medium and fetal bovine serum (FBS) from Biowest (Nuaillé, France) and XTT test from Biotium (Fremont, CA, USA). Bromophenol blue, dithiothreitol (DTT), glycerol, iodoacetamid, Immobiline Dry Strip gels, pH 4–7, Precast 12.5% polyacrylamide gel and buffer for 2-D DIGE, sodium dodecyl sulfate (SDS), Tris-HCl, UREA, (GE Healthcare, Little Chalfont, UK). Refraction-2D Labeling Kit and DyeAgnostics was obtained from LKB Biotech, Warsaw, Poland. All other reagents were obtained from POCH SA (Gliwice, Poland). Standard polystyrene flasks (T-75 flasks) and flat bottom cell culture microplates (12, 24, 48 and 96 wells) were from TPP Techno Plastic Products AG (Trasadingen, Switzerland). All other disposables were from VWR Int. (Gdansk, Poland).

### 2.3. Preparation of Biomaterial’s Samples

Compliance with the relevant [10,11] standards regarding composition of Ti6Al4V ELI alloy and PEEK used in this study was confirmed on the basis of certificates provided by the material manufacturers, which confirmed that they were medical grade materials intended for the production of implantable devices. Ti6Al4V alloy samples that had been produced using selective melting with electron beam technology (Ti6Al4V ELI-EBT) were also used in this study [12]. The samples were prepared in the form of discs (8 or 16 mm in diameter) and thickness of 3 mm. The surfaces of the samples were processed in accordance with the standards used for the production of implantable medical devices by LfC Ltd. company (Zielona Gora, Poland). Medical steel samples (American Iron and Steel Institute (AISI) 316L)—internal standard—were ground and polished. The final stages of sample preparation, including washing, double-sleeve packaging and steam sterilization were conducted in a clean zone.

### 2.4. Cell Culture

Human osteosarcoma cell line Saos-2 was cultured in McCoy’s 5A medium supplemented with 15% FBS together with penicillin and streptomycin. The cells were cultured under 98% humidity and 5% CO_2_ at 37 °C in standard polystyrene flasks (T-75 flasks). The medium was changed every 48 h and the cells were transferred to new flasks when confluence above 85% was reached.

### 2.5. Cytotoxicity Test

Materials for direct contact testing (16 mm in diameter) were placed in 12-well cell culture plates and then the Saos-2 cell line suspension was seeded at a number of 60,000 cells in 2 mL of the appropriate medium. The culture was maintained for 48 h at 37 °C, 5% CO_2_ and 98% humidity. After 48 h, the XTT test was carried out according to the manufacturer’s instructions (Biotium No. 30007). Untreated cells cultured in medium were taken as negative control, whilst cells treated with 50% ethanol for 30 min were considered as positive control.

### 2.6. Bacterial Colonization Test

Samples for bacterial colonization (8 mm diameter) were placed into separate wells of the flat bottom microplate (200 µL) and were immersed in the medium containing NaCl (1%), bactopeptone (1%) and yeast extract (0.5%), pH 7.0. The medium was inoculated using approximately 2 × 10^3^ *E. coli* cells. The samples with cells were cultured at 37 °C for 24 h. After that the samples were removed from the growth medium and were extensively washed out with deionized water. In the next step, the samples were fluorescence stained with bis-benzimide which stains living bacterial cells blue and with propidium iodide staining dead cells red. Each surface was soaked with the dyes by applying 20 µL of stock solution (100 mg/mL). The dye was allowed to penetrate the cells and this process was carried out in the dark, for 10 min at 28 °C. Finally, bacterial cells present on the sample surfaces were detected using a fluorescence microscope Olympus GX71 (Olympus, Tokyo, Japan) equipped with Charged Coupled Device (CCD) camera. Sample surfaces were analyzed in three separate and independent experiments and every surface was inspected in six randomly selected, but not overlapping areas. Experimental procedures have been described elsewhere [13,14].

### 2.7. Blood Platelets Adhesion Test

Blood was withdrawn from healthy volunteers who had not used any antiplatelet drugs for the past 2 weeks before the experiment. Sodium citrate (3.8%) was used as an anticoagulant. Each sample was incubated separately with the whole citrated blood at 37 °C for 1 h with gentle, end to end mixing. The samples were then rinsed with phosphate buffered saline, pH 7.4 and fixed in 2.5% glutaraldehyde for 1 h at 4 °C. After that, samples were rinsed again with PBS and dehydrated with ethanol used in gradually increasing concentration (50%, 70%, 80%, 90%, absolute alcohol). The dehydrated samples were observed with the use of fluorescence microscope Olympus GX71 (Olympus, Tokyo, Japan), scanning electron microscope HITACHI S-3000N (Hitachi, Tokyo, Japan), or both, after sputtering thin gold film in the sputtering apparatus JEOL JEE-4X (JEOL Ltd., Tokyo, Japan) [13]. Series of photographs of randomly selected areas were taken for each examined surface and a quantitative analysis of adhesion of platelets was performed with the use of Image J program [15]. Experimental procedures have been described elsewhere [16,17,18,19].

### 2.8. RNA and Protein Isolation

After 24 h cell culture on the sample surfaces, the cells were harvested with trypsin and lysed using TRI Reagent (Molecular Research Center) containing phenol and guanidine isothiocyanate. Lysis was carried out for 5 min at room temperature. The addition of chloroform and subsequent centrifugation at 12,000× *g* at 4 °C resulted in separation of the cell lysate into three layers: upper water layer containing RNA, middle layer with DNA and lower organic layer containing proteins. Isopropanol (Chempur, Poland) was used to precipitate RNA and proteins in separated tubes. Total RNA from Saos-2 cells was purified using RNeasy Mini Kit (Qiagen, Germantown, MD, USA) according to manufacturer’s instructions. Total RNA concentration was measured using NanoVue Plus spectrophotometer (GE Healthcare, Little Chalfont, UK). RNA quality and integrity was assessed by microfluidic capillary electrophoresis using an RNA 6000 Nano Kit (Agilent, Santa Clara, CA, USA) and 2100 Bioanalyzer (Agilent, Santa Clara, CA, USA). All RNA samples had RIN values higher than 8.0.

### 2.9. Microarray Analysis

Total RNA samples (200 ng) isolated from the cell culture were used to synthesize double-stranded cDNA and cyanine labeled cRNA using Quick Amp Labeling Kit (Agilent, Santa Clara, CA, USA) according to manufacturer’s instructions. Sample cRNA was labeled with cyanine 5-CTP and control cRNA was labeled with cyanine 3-CTP using T7 RNA polymerase in vitro Transcription Kit (Agilent, Santa Clara, CA, USA). Samples were purified using RNeasy Mini Kit (Qiagen, Germantown, MD, USA) according to manufacturer’s protocol. Each experimental cRNA sample was mixed with equimolar amounts of control cRNA and hybridized using Agilent SuperPrint G3 Human GE 8 × 60 K V2 Oligonucleotide Microarrays for 17 h in a hybridization chamber at 65 °C and a rotation speed of 10 rpm. Arrays were washed according to manufacturer’s protocol and scanned on a SureScan Microarray Scanner (Agilent, Santa Clara, CA, USA). Subsequently data from scanning images were extracted using Feature Extraction software v.11.0.1 (Agilent, Santa Clara, CA, USA). Gene Spring GX 14.5 software (ver. 14.5, Agilent, Santa Clara, CA, USA) was used to analyze gene expression data. The expression values were normalized according to median 75%, filtered with a flag tag to remove genes where expression levels were low and statistically analyzed using Tukey’s test with *p* < 0.05. Alterations in gene expression were represented as fold change ratio (FC) or log_2_FC (figures). The term “highly specifically altered expression” means altered expression at 0.5 > FC > 2 observed in cells exposed to the one particular biomaterial whereas for all other biomaterials FC = 1. The term “specifically expressed genes” means also altered expression at 0.5 > FC > 2 observed in cells exposed to the one particular biomaterial, but for at least one other biomaterial FC ≠ 1). Basic gene ontology analysis of differentially expressed genes (DEGs), in relation to biological processes, was performed with the use of bioinformatic tool Panther [19]. 

### 2.10. 2D Electrophoresis

Two-dimensional (2D) DIGE electrophoresis was employed for analysis of the whole proteome of the cells. Briefly, isolated and purified proteins (50 µg) were labeled with 400 pmol of Cy3 or Cy5 dyes in line with the protocol provided with Refraction-2D Labeling Kit. The internal standard was labeled with Cy2. Labeled proteins were mixed with rehydration buffer and applied onto 18 cm Immobiline Dry Strip gels, pH 4–7 and left in the dark at 20 °C for 12 h for the rehydration step. The first dimension of electrophoresis was run in IPGphor III (GE Healthcare, Little Chalfont, UK) at 20 °C overnight (minimum 60 kVh) and current of 50 μA per strip. Before the second dimension strips were equilibrated in a buffer containing 6 M urea, 75 mM Tris-HCl pH 8.8, 29.3% glycerol, 2% SDS and 0.002% bromophenol blue, supplemented with 1% DTT for 15 min and with 2.5% iodoacetamid for the next 15 min. Deionized water was used to prepare all solutions, Thermo Fisher Scientific Inc. (Waltham, MA, USA). In the next step, the strips were put on 12.5% polyacrylamide gels and placed in EttanDALTsix system (GE Healthcare, Little Chalfont, UK). The second dimension was run for about 6 h with the following parameters: 600 V and 400 mA and 20 W per gel. After completion of 2D electrophoresis, gels were visualized with a fluorescence scanner Typhoon FLA 9500 (GE Healthcare, Little Chalfont, UK). Changes in the proteome profile were analyzed with ImageMaster 2D Platinum 7.0 DIGE Software (GE Healthcare, Little Chalfont, UK).

### 2.11. Mass Spectrometry Analysis

The selected spots containing differently exposed fluorescently labeled proteins were excised from the gel, then washed twice with ammonium bicarbonate for 15 min, dehydrated with acetonitrile for 15 min and digested with 10 ng/mL of sequencing grade trypsin at 37 °C for 16 h (Ettan Digester, GE Healthcare, Little Chalfont, UK). Following digestion, tryptic peptides were extracted twice for 20 min with acetonitrile containing trifluoroacetic acid (TFA). Liquid chromatography-mass spectrometry (LC-MS/MS-Tandem mass spectrometry) analysis was performed with an integrated nanoLC-MS/MS system consisting of ESI-Ion Trap, AmaZon Speed ETD mass spectrometer (Bruker, Germany) fitted with nano-LC sprayer and liquid chromatography set Dionex Ultimate 3000 (Thermo Scientific), both operated under HyStar Software (Bruker, Germany). Injected samples were first trapped and desalted isocratically on Acclaim PepMap C18 precolumn (2 cm, 5 µm × 100 Å, Thermo Scientific), immediately after this peptides were eluted off and separated on an analytical C18 nano column (Acclaim PepMap C18, 15 cm, 3 µm × 100 Å) connected online to the mass spectrometer, at flow of 250 nL/min. A 90 min gradient of 5–90% acetonitrile with 0.04% of TFA was used. Proteins were identified in SwissProt database on in-house MASCOT server with the use of ProteinScape 3 Platform (Bruker, Germany).

### 2.12. Statistical Analysis

Statistical evaluation of the results was performed using a Shapiro–Wilk test to analyze the normality and one-way ANOVA with the post-hoc Tukey’s test for multiple comparisons and analysis of statistical significance between the average values. Values of *p* < 0.05 were considered statistically significant. Experiments were performed on cells from three independent cultures.

## 3. Results

### 3.1. Cytotoxicity Test

**Figure 1:**
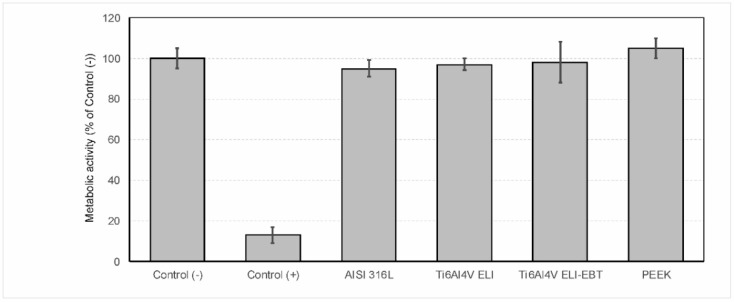
Results of cytotoxicity test assessed with cell metabolic activity. Negative and positive controls are described in the Section 2 (*n* = 3). PEEK, polyether ether ketone.

A medical steel AISI 316L sample was included in the study as the internal standard of our laboratory. As can be seen in Figure 1 none of the tested materials showed altered cytotoxicity.

### 3.2. Bacterial Colonization Test

**Figure 2:**
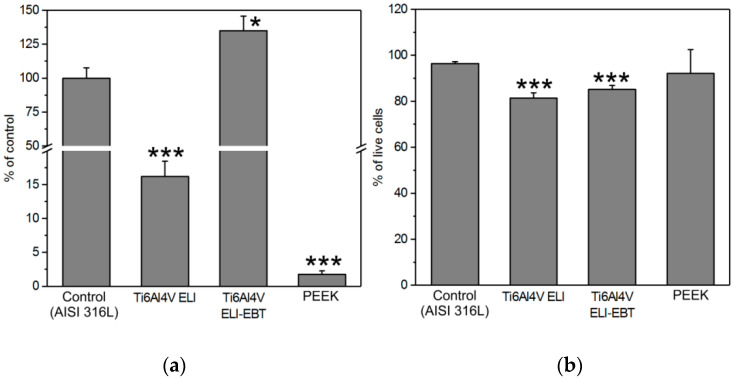
Susceptibility to microbial colonization of sample surface. (**a**) The total number of *Escherichia coli* cells found on the surface as a percent of the control. (**b**) The percentage of alive cells found on the surfaces (*n* = 3). * and *** indicate statistically significant differences vs. control for *p* < 0.05, *p* < 0.001, respectively.

In the next step, the tested materials were evaluated for susceptibility to colonization with microorganisms. As shown in Figure 2 the surfaces of both Ti6Al4V ELI and PEEK exhibit a significantly higher resistance to colonization with *E. coli* cells, while the more porous surface of the same titanium alloy produced by EBT technology is more susceptible to microbial colonization than the control surface of polished medical steel. None of the tested materials showed high toxicity in relation to *E. coli* cells, however about 15–20% of bacterial cells were designated as dead on the surfaces of both the titanium alloys, while on the surface of medical steel and PEEK this was only between 3 and 9%, respectively.

### 3.3. Blood Platelets Adhesion Test

**Figure 3:**
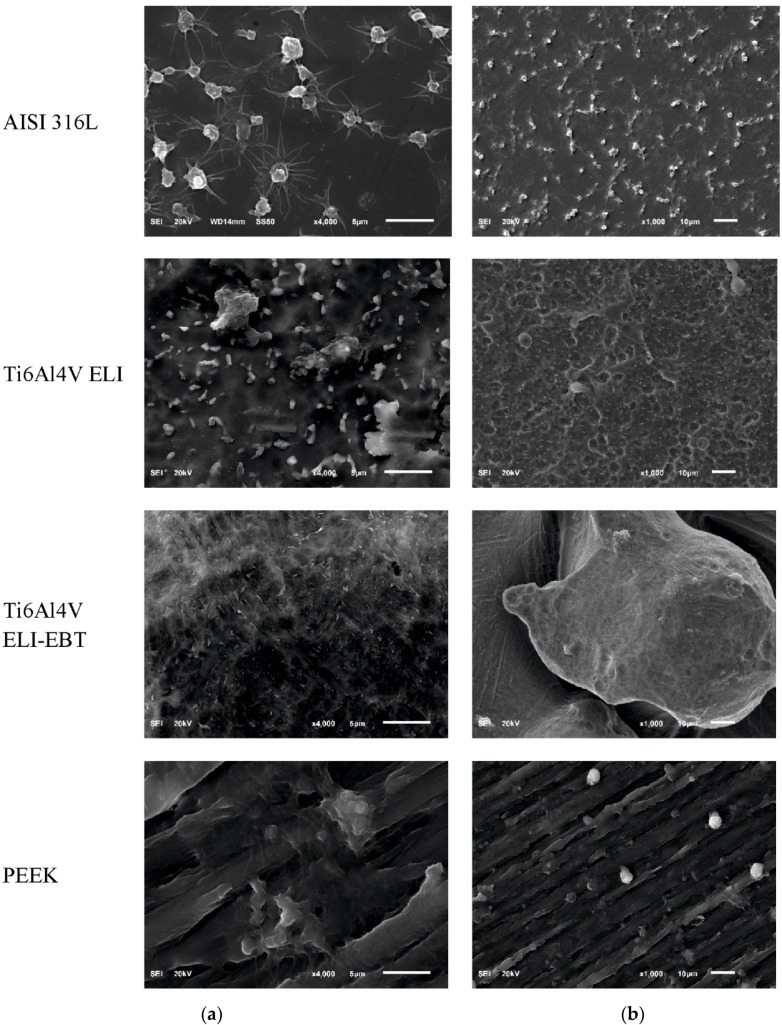
Example scanning electron microscopy (SEM) photographs of the tested surfaces after contact with blood. (**a**) 1000× (scale bar: 10 µm) and (**b**) 4000× (scale bar: 5 µm).

Platelet adhesion to the examined surfaces as well as their activation and aggregation were varied. Figure 3 shows randomly selected SEM photographs of the analyzed surfaces after contact with blood.

The number of adhered platelets in the form of singular platelets, small aggregates and large aggregates is presented in Table 1. Susceptibility to platelet adhesion was very high for polished medical steel AISI 316L, while much lower for the other biomaterials and can be ranked from lowest to highest—PEEK, Ti6Al4V ELI and Ti6Al4V ELI-EBT.

### 3.4. Microarray Analysis

**Figure 4:**
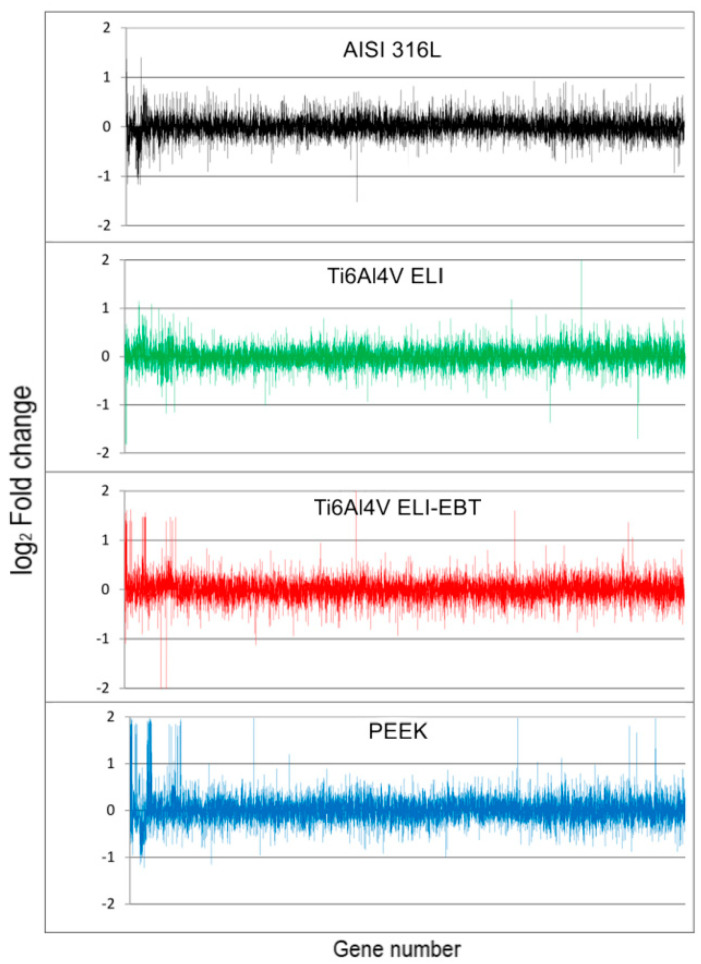
The gene expression profiles of cells exposed to medical steel AISI 316L, PEEK, Ti6Al7V ELI and Ti6Al7V ELI-EBT. Gene numbering from 1 to 58,319 according to their position on the Agilent SuperPrint G3 Human GE 8 × 60 K V2 Oligonucleotide Microarray.

Cells grown on the surfaces of the tested samples were lysed to analyze their transcriptome and proteome profiles. Isolated RNA was used to analyze gene expression profiles using the microarray technique, whereas isolated proteins and peptides were used for proteome profile analysis with 2D-DIGE separation and mass spectrometry peptide identification. Figure 4 illustrates a comparison of the expression profiles of active genes in cells separately exposed to the analyzed biomaterials.

Table 2 summarizes results of the microarray examination. Differential analysis of gene expression was employed, in which expression of all genes in the cells exposed to the biomaterial samples (S—sample cells gene expression) was compared to the same gene expression in control cells (C—control cells gene expression). For simplicity, the fold change ratio of sample cells gene expression to control cells gene expression (FC = S/C) was analyzed. It was assumed that a twofold change in gene expression (0.5 ≥ FC ≥ 2) was relevant. The number of expressed genes in Saos-2 cells exposed to contact with the examined biomaterials reached 9463 genes in total (ranging from 8455 genes expressed in cells exposed to ELI to 9160 genes in cells exposed to PEEK). Most genes were similarly expressed in cells exposed to contact with each and all of the biomaterials (within the range 0.5 ≤ FC ≤ 2); however, certain genes were overexpressed (19 genes, FC ≥ 2) or suppressed (6 genes, FC ≤ 0.5) as a result of cell exposure to one particular biomaterial—we refer to this as highly specific expression, whilst at the same time FC for all the other biomaterials was irrelevant. On the other hand, some genes were overexpressed (16 genes, FC ≥ 2) or suppressed (12 genes, FC ≤ 0.5) less specifically for more than one but not for all biomaterials and in these cases FC was also relevant—we refer to this as specific expression.

In Supplementary Materials, Table S1 contains the whole list of genes with highly specific and specific altered expression, up and down regulation notation, corresponding protein/transcript name, a short description of protein/transcript function and an expected metabolic pathway.

**Figure 5:**
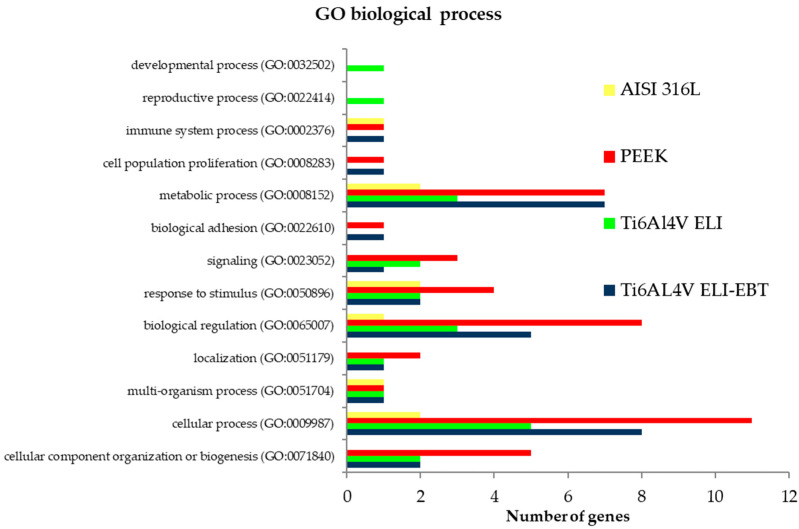
Gene ontology classification of differentially expressed genes (DEGs), in relation to biological processes.

With regard to differentially expressed genes (DEG) in cells exposed to all tested biomaterials, gene ontology analysis was employed to specify the biological processes affected by altered gene expression. Common classification of all DEGs is presented in Figure 5. The representative pathways refer to basic cellular functionality for example cell metabolism, cell proliferation, cell adhesion, signaling or response to stimuli. The most distinguished biological process was related to the category “cellular process” that includes cellular component organization, cell communication, cell death, export of substances from the cell or response to stress. In general, all tested biomaterials caused changes in specific molecular pathways, while the highest number of genes involved in a particular process was apparent for PEEK. It should be also stressed that among DEGs, some long non-coding RNAs and small nucleolar RNAs were found for each biomaterial.

**Figure 6:**
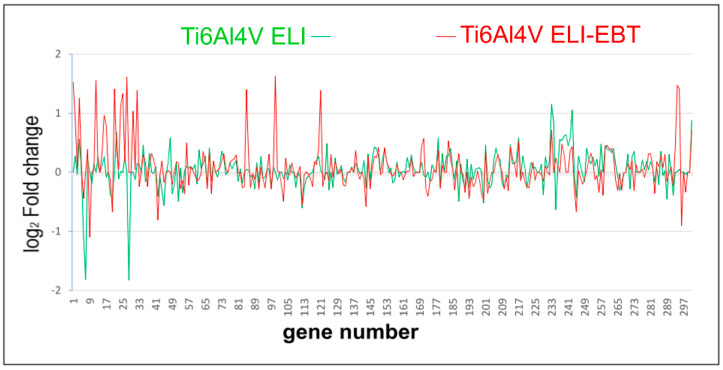
Comparison of a short set of example gene expression profiles obtained for cells exposed to samples made from the same titanium alloy Ti6Al4V, but produced using CNC machining (ELI) or selective melting with electron beam technology (ELI-EBT). Gene numbering according to their position on the Agilent SuperPrint G3 Human GE 8 × 60 K V2 Oligonucleotide Microarray.

Figure 6 shows the gene expression profiles of the first 297 genes, in order to compare the gene expression profiles obtained, for cells exposed to samples made from the same titanium alloy Ti6Al7V but produced using different manufacturing methods. The samples were produced by CNC machining (ELI) or selective melting with electron beam technology (ELI-EBT). Genes with similar gene expression profiles were more frequent than genes with altered expression. According to the adopted criterion, a significant change in gene expression applies to those genes for which log_2_ FC is greater than 1 or less than −1.

### 3.5. 2D Electrophoresis and Mass Spectrometry Analysis

Isolated proteins and peptides were analyzed by means of 2D-DIGE technique. It was found that several peptides/proteins were in altered expression which is presented in Table 3.

Due to a limited amount of peptides present in excised spots not all peptides could be identified with mass spectrometry. For the identified peptides, the name of the coding gene is also presented.

**Figure 7:**
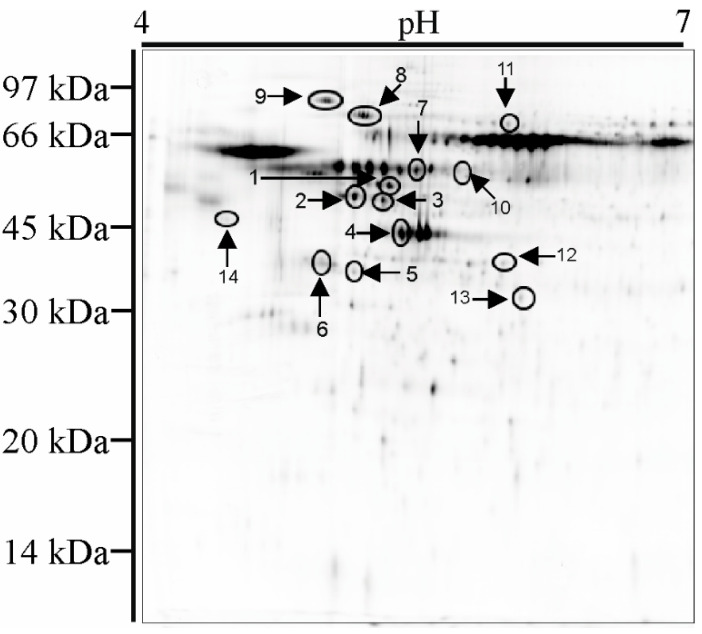
Representative 2D gel image of isolated and separated peptides from osteoblast cells. The outlines and numbers show the proteins identified by LC-MS/MS and presented in Supplementary Table S2.

An example gel with indicated spots containing differently expressed and identified peptides is presented in the Figure 7. Proteins present in 14 spots with altered expression were identified using mass spectrometry.

The complete list of identified proteins is provided in Supplementary materials Table S2. The list contains information about the name of the gene encoding the identified peptide, protein name, molecular weight (MW) and isoelectric point (pI) of the identified peptide, parameters confirming its identification (scores, number of identified peptide fragments and percent of protein sequence coverage), a brief description of the function of the protein as well as expected metabolic pathway.

## 4. Discussion

Metal and polymer biomaterials are commonly used in many types of surgery, including spine and orthopedic surgery. Despite the availability of a large variety of biomaterials, the ones most commonly used are titanium and its alloys as well as polymer biomaterials such as polyethylene and polyether ether ketone (PEEK), due to their optimal physicochemical properties. In general, these biomaterials meet the normative properties set out in the relevant international standards and are considered safe. However, with regards to the principles of materiomics there is a need for their broader characterization that additionally takes into account the molecular metabolic response of cells coming into contact with these biomaterials.

### 4.1. Cytotoxicity Test and Bacterial Colonization Test

Our comprehensive biological analysis of biomaterials that are commonly used in spine and orthopedic surgery shows that results of standard cytotoxicity tests, although consistent with data available in literature, do not provide complete information about the suitability of these materials. It should be noted that the porous surface structure of Ti6Al4V ELI-EBT promotes microbial colonization of this biomaterial. PEEK polymer looks better in this respect, although machined Ti6Al4V is also significantly less susceptible to microbial colonization when compared to the polished surface of AISI 316L medical steel. It should also be noted that the viability of *E. coli* cells is statistically significantly lower on titanium alloy surfaces.

### 4.2. Blood Platelets Adhesion Test

Sufficient availability of growth factors is very important for the integration of the implant with bone tissue. Activated platelets are a rich source of growth factors and in this sense the ease of adhesion, activation and aggregation of platelets on the surface of a material is an important parameter that should be effectively considered when choosing a biomaterial. PEEK polymer surface and machined Ti6Al4V surface exhibited a similar susceptibility to platelet adhesion and activation but were significantly less susceptible in comparison with the polished surface of AISI 316L medical steel, which is an in-house reference sample in our laboratory. The seemingly lower susceptibility of the titanium alloy surface obtained by EBT method is most likely due to the fact that it is very irregular and as a result we were not able to observe platelets that were most probably within porosities, to which blood had access.

### 4.3. Microarray Analysis

Contact of all the tested biomaterials with osteoblasts resulted in a change in the expression of numerous genes. Figure 4 shows a comparison of the full gene expression profiles in cells exposed to individual biomaterials. Due to the large number of genes (approximately sixty thousand) analyzed in one test, the presented results are for informative purposes only. A brief explanation of why such a large number of genes were analyzed is necessary, since the results of the Human Genome Project showed that the human genome contains between 20 and 30 thousand genes encoding proteins [20]. However, after completion of the Human Genomics Project it became apparent that different gene variants should also be analyzed, as well as non-coding genes and RNA coding genes that are important for cell function [21]. Consequently, almost 60,000 specific DNA fragments of the human genome in total are now represented in commercially available microarrays. Table 2 summarizes the entire number of active genes in Saos-2 cells exposed to the studied biomaterials, as well as the total number of active genes detected in the whole experiment. The lowest number of active genes was found for cells exposed to titanium alloy Ti6Al4V-ELI, and the highest number for PEEK. The authors arbitrarily assumed that only genes with a twofold change in expression, as compared to the control, exhibit a significant change in expression. The overwhelming majority of active genes in cells that were exposed to the tested biomaterials did not differ in expression in comparison to the control cells. However, some of the genes differed in expression both in relation to the control cells and between cells exposed to individual biomaterials. It has been previously reported that cells are able to recognize and respond to stress resulting from contact with different artificial surfaces in a highly specific manner [22,23,24]. Analysis of the results obtained in this study confirms this phenomenon and brings to light several new observations. There were genes with altered expression induced by cell contact with a specific biomaterial; however, these genes showed no change in expression in cells exposed to the other biomaterials being tested. We referred to this change in expression as highly-specific. In addition, there were changes in gene expression in cells exposed to one of the biomaterials together with altered expression of that same gene in cells exposed to the other biomaterials, however in the latter case the value of the change in expression was not significant (1 < FC < 2), we referred to this change in expression as specific. Table 2 presents a list of genes showing highly-specific and specific changes in gene expression divided into overexpression and suppression. It is obvious that exposure of Saos-2 cells to PEEK causes the most extensive changes in gene expression. A more detailed analysis of genes that meet the conditions for highly-specific and specific changes in expression is provided in the Supplementary Table S1. From this table the name of the product of gene expression can be seen, its function and the expected metabolic pathway in which it takes part. The ability of the cell to make a specific metabolic response following contact with an artificial surface is depicted in Figure 6 in which expression of 297 genes was compared, in order of their arrangement on microarrays, in cells exposed to Ti6Al4V ELI and Ti6Al4V ELI-EBT. The chemical composition of both alloys is the same, however the manufacturing methods used to produce the samples used in the study differ greatly and hence they have different surface structures. The similarity of the gene expression pattern in both cases is clearly visible. On the other hand, there were also specific differences that were clearly visible against the backdrop of a common pattern. The comparison was similar in all other areas of gene expression pattern and it confirms the existence of both specific and nonspecific cell responses to the stress resulting from exposure to artificial surfaces, which has been reported previously by the authors [22,23,24] and by others [25,26]. Unfortunately when taking into account transcriptomic analysis of cell response towards biomaterials, in particular titanium alloys, or PEEK, in current literature there is a lack of reports on this topic. We found only one paper by Sagomonyants et al. [27] where the authors examined in vitro response of osteoblast cells to different forms of PEEK material with the use of the real-time PCR technique for gene expression analysis. Three transcripts involved in bone formation, namely human alkaline phosphatase, Type I collagen and osteocalcin where taken into investigation. It was shown that the type of PEEK substrate modification can cause altered expression of tested genes in human osteoblasts. In our study, the corresponding gene transcripts where not affected for Ti6Al4V ELI and Ti6Al4V ELI-EBT, nor for PEEK after contact of Saos-2 cells with tested biomaterials. Another publication on the biological response of osteoblasts to contact with the PEEK polymer and pure titanium focused on the analysis of changes in the proteome of MG-63 cells after a specified culture time [28]. Although the work did not deal with transcriptomic analysis directly, it proved that specific metabolic pathways in the cell, in which differentially expressed proteins are involved, become affected after contact of cells with a given surface, which was also shown in our work.

### 4.4. 2D Electrophoresis and Mass Spectrometry Analysis

Due to the fact that not all gene expression translates into the creation of a gene product, namely, a peptide, studies on changes in the proteome profile of cells exposed to the tested biomaterials were also carried out. Table 3 shows the total number of detected spots for Saos-2 cells exposed to individual biomaterials, as well as the number of spots in altered expression and the names of proteins identified by mass spectrometry. Far more downregulated than upregulated proteins were found. From a group of spots with altered protein expression we were able to efficiently identify proteins for 14 spots only. Two proteins were suppressed in all cells exposed to all the studied biomaterials. We call these altered expressions nonspecific and common for all the studied biomaterials. One protein was upregulated in two cases, one protein was downregulated in three cases and additionally three proteins were downregulated in two cases. The other identified proteins were altered in a specific manner. The detailed analysis of identified proteins is provided in Supplementary Table S2. From this table the name of the encoding gene and the name of the protein can be seen, as well as their function and the expected metabolic pathway in which it takes part. It is worth noting that no pairs of altered genes—altered products of expression—were found. The explanation for this observation has been discussed extensively in our previous paper [23]. Molecular regulation together with control of translation and transcription processes, are responsible for a significant reduction in translation relative to transcription. In addition, prior RNA synthesis (transcription) is necessary for peptide synthesis (translation). These processes are always shifted in phase and our end-point analysis concerns the current content of transcripts and proteomes. Transcripts determine the future metabolic state of a cell whereas the proteome is a result of its current metabolic state and reflects the state of gene expression in the past. Supplementary Table S3 lists the metabolic pathways in which the identified genes and proteins manifest their metabolic activity. Despite the apparent chaos and lack of correlation between transcripts and proteomes, which we could expect to see, the data presented in this table do in fact exhibit a certain degree of order. As shown, twelve out of the fourteen metabolic pathways resulting from altered gene expression and twelve out of the twenty-one metabolic pathways resulting from altered protein expression are the same. This strongly suggests that the metabolic response of cells does not necessarily have to be regulated by the same genes and proteins. More importantly, it is the activation of appropriate metabolic pathways, which have been demonstrated by the results presented, that is crucial to the proper functioning of a cell and its adaptation to stress. The most important metabolic pathways common to the transcriptome and proteome should certainly include those that are most represented, namely: cell metabolism, gene expression, immune system, signal transduction, disease control and developmental biology. Of course, the significance of metabolic pathways that are only represented by identified proteins or genes should not be underestimated. Among them, there are important pathways such as chaperone activity, vesicle mediated transport, cell cycle regulation and chromatin organization. The above emphasizes the complexity of processes triggered within cells subjected to stress and exposed to contact with biomaterial surfaces; these are processes that we do not fully understand yet, nor do we appreciate their significance based on our current state of knowledge, particularly in the context of the rapidly approaching era of personalized medicine, which will require selection of specific biomaterials best suited to the individual genetically and environmentally conditioned needs of the patient. It is without doubt that the strategy introduced by materiomics brings us closer to the goal of production and effective use of implants in personalized medicine.

## 5. Conclusions

In summary, none of the biomaterials tested showed unsatisfactory levels of cytotoxicity, which was to be expected. However, susceptibility to microbiological colonization and susceptibility to adhesion, activation and aggregation of blood platelets differentiate these materials and allow for making a rational choice depending on the expected needs. The gene expression analysis performed, which represents a completely new approach to these biomaterials, shows that the PEEK polymer causes far more intense changes in gene expression and thus also cell metabolism. This indicates the need to look more closely at this highly regarded and commonly used biomaterial. It is particularly important to assess whether such extensive changes are permanent and result from triggering cell adaptation processes or whether they should be treated as an early metabolic response of the cell.

## Figures and Tables

**Table 1:** Blood platelet adhesion to the studied samples. The platelet number was determined by the use of photos obtained with scanning electron microscopy (SEM) and corresponds to the number per 3–10 mm^2^. At least 10 separate photos were analyzed for each material.

Biomaterial	AISI 316L	PEEK	Ti6Al4V ELI	Ti6Al4V ELI-EBT
small aggregates (<10 platelets)	34.8 ± 14.6	2.3 ± 1.1	1.7 ± 0.9	0.65 ± 1.0
large aggregates (>10 platelets)	3.7 ± 3.5	1.0 ± 1.3	1.4 ± 0.8	0.2 ± 0.5
singular platelets	28.8 ± 16.2	3.5 ± 3.7	0.0 ± 0.0	0.6 ± 0.7

**Table 2:** The number of active genes and the number of genes with altered expression as well as the number of specifically expressed genes at 0.5 ≥ Fold Change (FC) ≥ 2.

Biomaterial	AISI 316L	PEEK	Ti6Al4V ELI	Ti6Al4V ELI-EBT
Number of expressed genes	8716	9160	8455	8975
Number of highly specifically overexpressed genes for one particular biomaterial at FC ≥ 2, and FC irrelevant for others.	1	14	3	1
Name of genes	VAC14	PMCH	XLOC_l2_008203	lnc-TNFRSF14-2
		GPR182	SPAG9	
		UQCRFS1	PRR5-ARHGAP8	
		SNX18		
		GFRA2		
		LOC102723429		
		MAP2K6		
		lnc-DYDC1-4		
		A_22_P00017766		
		lnc-ZC3H12D-2		
		A_22_P00022299		
		LOC101928894		
		DHRS4L1		
		PROM2		
Number of highly specifically suppressed genes for one particular biomaterial at FC ≤ 0.5, and FC irrelevant for others.	0	1	3	2
Name of genes	-	TOMM20L	SCRG1	A_22_P00001871
			USP14	LOC100507053
			POMZP3	
Number of specifically overexpressed genes for one particular biomaterial at FC ≥ 2, and FC also relevant for at least one but not all other.	3	9	3	1
Name of genes	HMOX1	TMEM158	SNORA16A	TMEM65
	G6PD	SMARCA4	SNORD105B	
	RNU6ATAC	MMP14	lnc-RP1-177G6.2.1-2	
		MTRNR2L2		
		lnc-MINA-3		
		lnc-RNF13-2		
		HS3ST1		
		ADAMTS1		
		IL6ST		
Number of specifically suppressed genes for one particular biomaterial at FC ≤ 0.5, and FC also relevant for at least one but not all other.	3	2	5	2
Name of genes	IFIT1	SNORD12C	TAF15	CPA4
	SNORA48	APH1A	FOXD3-AS1	ANKRD1
	SNORA2B		A_22_P00020320	
			PPA2	
			NLE1	

**Table 3:** The number of up and down regulated proteins in Saos-2 cells exposed to the studied biomaterials. Due to a limited amount of peptides present in excised spots not all peptides could be identified with mass spectrometry. For the identified peptides, the name of the coding gene is also presented.

	AISI 316L	PEEK	Ti6Al4V ELI	Ti6Al4V ELI-EBT
Number of detected spots	739	755	656	700
Upregulated proteins				
Number of proteins	29	27	33	14
Identified proteins gene name		ALBU	ALBU	
Downregulated proteins				
Number of proteins	131	114	190	119
Identified proteins gene name	ACTB	ACTB	ACTB	ACTB
	RLA0	RLA0	RLA0	RLA0
	RSSA	RSSA	LDHB	RSSA
	UCHL1	UCHL1	ENPL	
	LDHB		ATPB/PDIA6	
	ENPL		CH60/HNRPK	
	HS90A		HNRPK	
	HS90B		TBB5	
			TBA1B	

## Supplementary Materials

The following are available online at https://www.mdpi.com/1996-1944/13/21/4769/s1, Table S1: List of genes with highly specifically and specifically altered expression. Up (↑) and down (↓) regulation is indicated. Protein/Transcript function collected and compiled from https://www.ncbi.nlm.nih.gov/ and https://www.uniprot.org/ databases. Metabolic pathways were collected and compiled from https://reactome.org/ and https://www.genecards.org/ databases, Table S2: List of differentially expressed proteins identified with LC-MS/MS. Protein function collected and compiled from https://www.uniprot.org/ database. Metabolic pathways were collected and compiled from https://reactome.org/ and https://www.genecards.org/ databases, Table S3: Expected metabolic pathways assigned to results obtained on the base of transcriptome and proteome analyses. The frequency of the metabolic pathway representation is given in brackets.
