# Supplementary material for: Comprehensive Biological Evaluation of Biomaterials Used in Spinal and Orthopedic Surgery
Source: Materials (Basel). 2020 Oct 26;13(21):4769. doi: 10.3390/ma13214769 (PMC7672648; doi:10.3390/ma13214769)
Supplement: Supplementary file 1 [file materials-13-04769-s001.pdf]

materials

Supplementary materials

Comprehensive Biological Evaluation of Biomaterials Used in Spinal and Orthopedic Surgery

Piotr Komorowski [1,2,*], Małgorzata Siatkowska [1], Marta Kamińska [2], Witold Jakubowski [2], Marta Walczyńska [2,3], Magdalena Walkowiak-Przybyło [2], Witold Szymański [2], Katarzyna Piersa [1], Patryk Wielowski [1], Paulina Sokołowska [1,4], Kamila Białkowska [1,5], Krzysztof Makowski [6], Marcin Elgalal [1,7], Agnieszka Kierzkowska [8], Lechosław Ciupik [8] and Bogdan Walkowiak [1,2,6]

1 Molecular and Nanostructural Biophysics Laboratory, "Bionanopark" Ldt., Dubois 114/116,93-465 Lodz, Poland; m.siatkowska@bionanopark.pl (M.S.); k.dzialoszynska@bionanopark.pl (K.P.); wielowskipatryk@gmail.com (P.W.); p.sokolowska@bionanopark.pl (P.S.); k.bialkowska@bionanopark.pl (K.B.); m.elgalal@bionanopark.pl (M.E.); bogdan.walkowiak@p.lodz.pl (B.W.)
2 Division of Biophysics, Institute of Materials Science, Lodz University of Technology, Stefanowskiego 1/15, 90-924 Lodz, Poland; marta.kaminska@p.lodz.pl (M.K.); witold.jakubowski@p.lodz.pl (W.J.); marta.walczynska@p.lodz.pl (M.W.); magdalena.walkowiak-przybylo@p.lodz.pl (M.W.-P.); witold.szymanski@p.lodz.pl (W.S.)
3 Department of Medical Imaging Technique, Medical University of Lodz, Lindleya 8, 90-419 Lodz, Poland
4 Department of Pharmacology and Toxicology, Medical University of Lodz, Zeligowskiego St. 7/9, 90-752 Lodz, Poland
5 Department of General Biophysics, University of Lodz, Pomorska 141/143, 90, 90-236 Lodz, Poland
6 Industrial Biotechnology Laboratory, "Bionanopark" Ldt., Dubois 114/116, 93-465 Lodz, Poland; k.makowski@bionanopark.pl
7 Department of Diagnostic Imaging, Radiation and Isotope Therapy, Medical University of Lodz, Pomorska 251, 92-215 Lodz, Poland
8 "LfC" Ltd., Kozuchowska 41, 65-364 Zielona Gora, Poland; a.kierzkowska@lfc.com.pl (A.K.); l.ciupik@lfc.com.pl (L.C.)
* Correspondence: piotr.komorowski@p.lodz.pl

Results.

Table S1 contains the whole list of genes with highly specifically and specifically altered expression, indication of up and down regulation, the corresponding protein/transcript name and a short description of protein/transcript function. Protein function was collected and compiled from https://www.ncbi.nlm.nih.gov/ and https://www.uniprot.org/ batabases. Metabolic pathways were collected and compiled from https://reactome.org/ and https://www.genecards.org/ databases.

Table S2. List of differentially expressed proteins identified with LC-MS/MS. Protein function collected and compiled from https://www.uniprot.org/ database. Metabolic pathways were collected and compiled from https://reactome.org/ and https://www.genecards.org/ databases.

Table S1. List of genes with highly specifically and specifically altered expression. Up (↑) and down (↓) regulation is indicated. Protein/Transcript function collected and compiled from https://www.ncbi.nlm.nih.gov/ and https://www.uniprot.org/ databases. Metabolic pathways were collected and compiled from https://reactome.org/ and https://www.genecards.org/ databases.

Sample	Gene Name (Human)	Up/Down Regulation	Protein/Transcript Name	Protein/Transcript Function	Expected Metabolic Pathways
**Highly Specifically Altered Expression**					
AISI 316L	VAC14	↑	VAC14 Homolog	A scaffold protein that is a component of the PIKfyve protein kinase complex which is responsible for the synthesis of phosphatidylinositol 3,5–bisphosphate, one of the seven phosphoinositides important for proper functioning of cellular membranes	Metabolism of lipids, Phospholipid metabolism, PIP Metabolosm (synthesis)
PEEK	PMCH	↑	Pro-MCH	Pro-MCH is proteolytically processed in the brain and in peripherial organs generating multiply protein products. Main neuropeptides are melanin-concentrating hormone (MCH), neuropeptide-glutamic acid-isoleucine (NEI), and neuropeptide-glycine-glutamic acid (NGE) that play diverse physiological functions, including energy homeostasis.	Signal transduction
	GPR182		G-protein coupled receptor 182	A seven-transmembrane protein that belongs to the family 1 of G-protein coupled receptors, probably acting as a receptor for adrenomedullin - a vasodilator peptide hormone expressed by all tissues, widely distributed in the cardiovascular system.	Sympathetic nerve pathway
	UQCRFS1		Cytochrome b-c1 complex mitochondrial subunit Rieske	A component of ubiquinol-cytochrome C reductase dimer complex. As a respiratory chain, this complex generates energy essential for ATP synthesis in mitochondria.	Metabolism, respiratory electron transport
	SNX18		Protein sorting nexin-18, containing phox domain	Members of sorting nexin family are important during progression and completion of mitosis. In case of any deficiencies in the expression of those proteins, the cytokinesis are inhibited and as a result the cell cannot finish the division. SNX18 protein was also identified as a positive regulator of autophagosome formation, that during macroautophagy fuses with lysosome. Expendable cytoplasmic constituents are then degraded and recycled within the autolysosome.	Metabolism, respiratory electron transport Autophagy
	GFRA2		GDNF family receptor alpha-2	GDNF (glial cell line-derived neurotrophic factor family) that constitute glycosylphosphatidylinositol (GPI)-linked cell surface receptor for GDNF and NTN. Other important function of GDNF receptor alpha-2 is activation of RET tyrosine kinase receptor.	Metabolism, respiratory electron transport
	LOC102723429		Uncharacterized gene LOC102723429 situated on short arm of 16th chromosome	No further knowledge is available.	

MAP2K6		Dual specificity mitogen-activated protein kinase 6	Plays an important role in signal transduction pathway. It phosphorylates a threonine and a tyrosine residue in p38 MAP kinase during different kinds of cellular stress or in response to inflammatory cytokines. Changed activity of this class of proteins may result in higher activity of NF-κB—a transcription factor regulating balance between cell survival and cell death. Dysregulation of its function may result in activation of genes involved in cancerous processes. This gene is upregulated in different kinds of cancer.	Immune system, MAO kinase activation and Interleukin-1 family signalling<br>Cellular response system, oxidative stress induced senescence<br>Gene expression, regulation of transcription
lnc-DYDC1-4		RNA gene belonging to the long non-coding RNA class	This gene was found to be overexpressed in patients suffered from one of subtype of acute lymphoblastic leukaemia	Gene expression, regulation of RUNX1 expression and activity
A_22_P00017766		Not annotated	No further knowledge is available.	
lnc-ZC3H12D-2		RNA Gene belonging to the long non-coding RNA class	This gene expression was found to be characteristic for one of molecular subtype of breast cancer (subtype HER2 + HR+)	Gene expression, regulation of RUNX1 expression and activity
A_22_P00022299		Not annotated	No further knowledge is available.	
LOC101928894		Uncharacterized gene situated on short arm of 11th chromosome	No further knowledge is available.	
DHRS4L1		putative dehydrogenase/reductase SDR family member 4-like 1	Plays an oxidoreductase function but no further knowledge is available.	Signal transduction<br>Retinoic Acid biosynthesis pathway<br>Signalling by Retinoic Acid (RA)
PROM2		Prominin 2 a member of prominin family	Pentaspan membrane glycoprotein. It has five transmembrane segments. It is mainly expressed in epithelial cells. It binds cholesterol and plays a pivotal role in organization of membrane microdomains, such as membrane protrusions essential in diverse biological processes like cell migration and adhesion, wound healing or sensing external environment. It inhibits caveolae formation due to inhibition of CDC42 (involved in the organization of the actin cytoskeleton and endocytosis). Together with overexpression of prominin-2, larger number of membrane protrusions was observed as well as changes in PM organization.	
TOMM20L	↓	Translocase of outer mitochondrial membrane 20 like	Participate in assembly of translocase membrane complex responsible for passing of proteins into mitochondrial compartments. Large part of proteins needed for proper mitochondria function is encoded by nucleus of the cell thus those proteins must be transported through mitochondrial membrane to the intermembrane space. Mitochondrial membrane is impermeable for molecules larger than 5 kDa, thus proper functioning of TOM complex is crucial for mitochondria.	

ELI	XLOC_l2_008203	↑	RNA gene, belonging to the long non-coding RNA class.	No further knowledge is available.	
	SPAG9		C-Jun-N-terminal kinase-interacting protein 4 (other name: sperm associated antigen 9)	Belongs to the group of scaffold proteins mediating the activation of JNK signaling pathway that controls different cellular processes like proliferation or apoptosis. It was shown that overexpression of SPAG9 gene is present in different kinds of cancer including lung, ovarian, breast and gastric cancer. The protein product probably influence tumor growth and development however up to now, its exact role in this process is still obscure.	Developmental biology, Myogenesis
	PRR5-ARHGAP8		PRR5-ARHGAP8 readthrough	A fusion protein that contains sequences identical with proline rich 5, renal (PRR5) and Rho GTPase activating protein 8 (ARHGAP8) genes' products. Resulting protein participates in signal transduction but its exact role is still unknown.	Signal transduction by Rho GTPases<br>Gene expression,<br>Constitutive signaling in diseases
	SCRG1	↓	Stimulator of chondrogenesis 1	It is associated with neurodegeneration and may participate in host response to prion infections. It was shown that this protein may influence mesenchymal stem cells differentiation during bone regeneration by interacting with bone marrow stromal cell antigen 1	
	USP14		Ubiquitin specific peptidase 14	It release the ubiquitin from proteins designated to be transported to proteasome. It regulates the function of proteasome and have a pivotal role in the development of human carcinoma.	Metabolism of proteins, Deubiquitinination
	POMZP3		POM121 and ZP3 fusion protein	The protein participate in acrosin binding—a major proteolytic enzyme of mammalian spermatozoa, present in acrosome. This protein functions as a positive regulator of acrosome reaction, thus influencing the process of fertilization.	
ELI-EBT	lnc-TNFRSF14-2	↑	RNA gene affiliated with long non-coding RNA class	Tumor Necrosis Factor Superfamily Member	Immune system costimulation by CD28 family and TNFs bind their physiological receptors
	A_22_P00001871	↓	Not annotated	No further knowledge is available.	
	LOC100507053		Uncharacterized gene situated on a long arm of 4th chromosome	No further knowledge is available.	
**Specifically Altered Expression**					
	HMOX1	↑	Heme oxygenase 1	It is responsible for heme metabolism. It cleaves the heme ring to form biliverdin, that is subsequently converted to bilirubin.	Transport of small molecules Iron uptake and transport<br>Immune system interleukin 4 and 13 signalling<br>Protein localization

AISI 316L	G6PD		Glucose-6-phosphate dehydrogenase	An cytosolic enzyme participating in pentose phosphate pathway, and responsible for NADPH production. Proper level of NADPH, an electron donor is important during oxidative response of the cell	Metabolism, pentose phosphate pathway
	RNU6ATAC		snRNA, U6atac small nuclear	Belongs to Lsm class of snRNA. snRNA function as a ribozymes during alternative splicing in the nucleus	
	IFIT1		Interferon induced protein with tetratricopeptide repeats 1	A protein that inhibits expression of viral mRNA thus inhibiting initiation of translation and replication during viral infection. This antiviral activity is characteristic against several types of viruses, including human papilloma and hepatitis C viruses.	Immune system, interferon alpha/beta signalling and antiviral signalling
	SNORA48	↓	Small nucleolar RNA, H/ACA box 48	Participates in pseudouridylation of rRNA, a process that influence ribosome functionality.	
	SNORA2B		Small nucleolar RNA, H/ACA box 2B	Is associated with specified proteins and functions as a ribonucleoproteins that have enzymatic activity towards rRNA, thus are crucial for such processes like protein synthesis, mRNA splicing or maintaining genome integrity	
	TMEM158		Transmembrane protein 158	It is acting as a receptor for brain injury-derived neurotrophic peptide (BINP). The role of BINP is to support of neuron survival and protection of hippocampal neurons in primary cultures from glutamate-induced cell death.	
	SMARCA4		Transcription activator BRG1	A protein that is a part of a large complex SNF/SWI and regulates the activation of certain genes by rearrangement of chromatin structure. This protein can play its function due to helicase and ATPase activity. What is more it can bind to BRCA1 and also influence the expression of tumorigenic protein CD44	Signal transduction,<br>Developmental biology, nervous system development<br>Gene expression by RUNX1<br>Chromatin organization
PEEK	MMP14	↑	Matrix metallopeptidase 14	A protein belonging to the MMP family involved in various physiological processes - e.g., tissue remodelling, embryonic development or in pathological conditions like arthritis and metastasis. It participates in collagen degradation, and also in skeletal connective tissue modelling. It was established that this protein activates MMP2 protein and together they are involved in formation of fibrovascular tissues. According to literature the activity of MMP2 is related to tumour invasion.	Extracellular matrix organization, collagen degradation, activation of matrix metalloproteinases
	MTRNR2L2		Humanin-like 2<br>a peptide of 24 amino acids	It was shown that this protein plays a neuroprotective role and suppress Alzheimer Disease-related neurotoxicity *in vivo*. It demonstrates an antiapoptotic activity of humanin *in vitro*—it inhibits the executive phase of apoptosis by interaction with Bax protein (an apoptosis-inducing protein) preventing its translocation from cytosol to mitochondria and thus - suppressing cytochrome C release.	
	lnc-MINA-3		RNA gene affiliated with long non-coding RNA class	Differential expression (down-regulation) of this long non-coding RNA was found in patients suffered from cirrhosis that ultimately can lead to hepatocellular carcinoma development.	Chromatin organization

	lnc-RNF13-2		RNA gene affiliated with long non-coding RNA class	No further knowledge is available.	Innate immune system
	HS3ST1		Heparan sulfate–glucosamine 3–sulfotransferase 1	Is present in Golgi apparatus and has a transferase activity that transfer sulfo group to glucosamine residue in heparan. Also plays role in anticoagulant heparan sulfate biosynthesis and conversion. Heparan sulfate is a linear polysaccharide that exhibit multiple biologic activities. It may bind with variety of protein ligands and form a proteoglycan (HSPG) that participates in regulating various biological processes, e.g., angiogenesis, blood coagulation, metastasis.	Metabolism, glycosaminoglycan metabolism
	ADAMTS1		ADAM metallopeptidase with thrombospondin type 1 motif 1	A protein belonging to the ADAMTS family, having in its structure metalloproteinase domain, disintegrin-like domain, and thrombospondin-type motif. Exhibit antiangiogenic activity, expressed during process of inflammation; is also involved in development of cancer cachexia.	Metabolism of proteins, glycosylation<br>Extracellular matrix organisation<br>Degradation, collagen synthesis and modification,<br>Disease (Peters-plus syndrome)
	IL6ST		Interleukin 6 signal transducer	A part of membrane-bound cytokine receptor complex. Acts as a signal transducer for IL6, LIF, OSM, CNTF, IL11, CTF1 and BSF3. Binding a substrate causes homodimerization of IL6SST which in turn activate Janus kinase, that phosphorylates tyrosine residues on Il6ST and finally activates STAT3 (transcription activator).	Immune system interleukin 6 and 12 signalling
	SNORD12C		Small nucleolar RNA, C/D box 12C	It contains C (UGAUGA) and D (CUGA) box sequence motifs that are highly conserved. The main function of snRNAs from box C/D family is to participate in process of RNA methylation. SNORD12C is predicted to play role during 2′ O-ribose methylation of the ribosomal 28S RNA subunit.	
	APH1A	↓	Aph-1 homolog A, gamma-secretase subunit	That is a non-catalytic component of gamma secretase complex (endoprotease complex) responsible for cleavage of integral membrane proteins playing important role in Notch or Wnt signalling cascades. It participates also in APP (amyloid-beta precursor protein) processing. It was shown that APH-1A gene polymorphism contribute to increased incidence of Alzheimer Disease.	Signal transduction by NOTCH1, 3, 4,
ELI	SNORA16A	↑	Small nucleolar RNA, H/ACA box 16A, 134 nucleotides long	snRNAs with H/ACA box were identified as factors participating in pseudouridynylation of 18S and 28S rRNAs, influencing a ribosomal functional features.	
	SNORD105B		Small nucleolar RNA, 92 nucleotides long, C/D box 105B	Can acts as oncogene and is involved in development of gastric cancer.	
	lnc-RP1-177G6.2.1-2		Long non-coding RNA	No further knowledge is available.	Gene expression
	TAF15	↓	TATA-binding protein-associated factor 2N	It belongs to a TET family of RNA-binding proteins. It participates in the process of gene transcription by polymerase II as a component of TFIID complexes.	Gene expression regulation<br>Diseases, infectious<br>Chromatin organization

	FOXD3-AS1		Non-coding RNA: FOXD3 antisense RNA 1	It was shown that downregulation of FOXD3-AS1 in malignant glioma cells has an inhibitory effect on cell proliferation, migration and invasion. Upregulation of this RNA gene results in poorer prognosis and lower survival probability for glioma patients. It was stated that FOXD3-AS1 has a tumorigenic property that may cause development of glioma. Another study demonstrated that this non-coding RNA is highly expressed in breast cancer—and may serve as a prognostic biomarker for breast cancer patients.	Developmental biology, transcriptional regulation of stem cells
	A_22_P00020320		Not annotated	No further knowledge is available.	
	PPA2		Inorganic pyrophosphatase 2	A protein present in mitochondria that catalyze the pyrophosphate hydrolysis to the inorganic phosphate. Impaired function of this hydrolase can result in deregulation of mitochondrial membrane potential, affecting proper function of whole mitochondrium. It is also essential for proper cell metabolism. Mutation in PPA2 gene is correlated with occurrence of mitochondrial diseases and cardiomyopathy.	Metabolism, phyrophosphate hydrolysis
	NLE1		Notchless homolog 1	A protein that takes part in regulation of CDKN1A expression and other proteins involved in Wnt signal transduction pathways. In Wnt signaling pathways signals are passed to the cell through cell surface receptors and are important for normal cell functioning, but also impaired activity of this pathway can contribute to cancer spreading.	
ELI-EBT	TMEM65	↑	Transmembrane protein 65	A mitochondrial inner-membrane protein. It was demonstrated that this protein plays a significant role in mitochondrial respiratory chain. Mutation in TMEM65 results in mitochondrial dysfunction and clinically is demonstrated by encephalomyopathy. This protein is also suspected for participating in maintaining mitochondrial DNA stability.	Metabolism of lipids estrogen, cholesterol synthesis<br>Immune system, interleukin 3, 5 and GM-CSF signaling<br>Organelle biogenesis and maintenance<br>Cell-cell communication Nectin/Necl trans heterodimerization<br>Metabolism, complex I biogenesis and electron transport<br>Transport of small molecules
	CPA4	↓	Carboxypeptidase A4	A metalloprotease belonging to a carboxypeptidase A/B subfamily, that catalyses proteolysis of C-terminal amino acids. It plays a probable role in histone hyperacetylation and also is considered as a cancer related gene. Increased expression of CPA4 protein was found in cancer tissue derived from patients suffered from colorectal cancer. It was shown that the presence of elevated level of this protein was corelated with poorer prognosis. In vivo studies confirmed tumorigenic function of this protein due to activation of STAT3 and ERK pathways (the first one act as a signal transducer and activator of transcription, the later - is a mitogen activated protein kinase that regulates different cellular processes like autophagy,	

		protein trafficking and integrity of genome). What is more, group of Sun et al. has observed that higher expression of CPA4 was a good prognostic parameter for prediction of hepatic metastasis, its progression and stage.	
ANKRD1	Ankyrin repeat domain 1	A protein involved in activation of endothelial cells. It is localized in cell nucleus and may play role of transcription factor that regulates cardiac gene expression. It is also linked to the epithelial-mesenchymal transition (EMT) during cancer development.<br>It was shown that ANKRD1 is overexpressed in EGFR-TKIs-resistant non-small cell lung cancer (NSCLC; EGFR-TKIs - epidermal growth factor receptor tyrosine kinase inhibitors). ANKRD1 gene silencing caused decreased resistance of cancer cells towards therapeutic drugs—afatinib and osimertinib. Inhibition of ANKRD1 expression at mRNA or protein level can be a promising therapeutic target for EGFR-mutant NSCLC patients.	Metabolism, lipid metabolism

Table S2. List of differentially expressed proteins identified with LC-MS/MS. Protein function collected and compiled from https://www.uniprot.org/ database. Metabolic pathways were collected and compiled from https://reactome.org/ and https://www.genecards.org/ databases.

Spot No.	Gene Name (Human)	Full Protein Name	Molecular Weight [kDa]	Isoelectric Point [pI]	Scores	Number of Identified Peptides	Protein Sequence Coverage [%]	Protein Function (UniProt Database)	Expected Metabolic Pathways
1	TBA1B	Tubulin alpha-1B chain OS = Homo sapiens GN = TUBA1B PE= 1 SV = 1	50.1	4.8	474.3	10	27.9	Tubulin is the major constituent of microtubules. It binds two moles of GTP, one at an exchangeable site on the beta chain and one at a non-exchangeable site on the alpha chain.	Development,<br>Chaperon mediated protein folding<br>Cytoskeleton remodeling<br>Ubiquitin-Proteasomal Pathway<br>Cel cycle regulation<br>Development<br>Innate immune system<br>Organelle biogenesis and maintenance
2	TBB5	Tubulin beta chain OS = Homo sapiens GN = TUBB PE = 1 SV = 2	49.6	4.6	920.7	19	37.6		
3	ATPB	ATP synthase subunit beta, mitochondrial OS = Homo sapiens GN = ATP5B PE = 1 SV = 3	56.5	5.1	151.8	3	6.2	Mitochondrial membrane ATP synthase (F1F0 ATP synthase or Complex V) produces ATP from ADP in the presence of a proton gradient across the membrane which is generated by electron transport complexes of the respiratory chain. F-type ATPases consist of two structural domains, F1—containing the extramembraneous catalytic core, and F0 - containing the membrane proton channel, linked together by a central stalk and a peripheral stalk. During catalysis, ATP synthesis in the catalytic domain of F1 is coupled via a rotary mechanism of the central stalk subunits to proton translocation. Subunits alpha and beta form the catalytic core in F1. Rotation of the central stalk against the surrounding alpha3beta3 subunits leads to	Metabolosm,<br>respiratory electron transport<br>Organelle biogenesis and maintenance<br>Mitochondrial biogenesis,<br>mitochondrial protein import

								hydrolysis of ATP in three separate catalytic sites on the beta subunits.	
	PDIA6	Protein disulfide-isomerase A6 OS = Homo sapiens GN = PDIA6 PE = 1 SV = 1	48.1	4.8	114.6	3	9.3	May function as a chaperone that inhibits aggregation of misfolded proteins. Plays a role in platelet aggregation and activation by agonists such as convulxin, collagen and thrombin.	Metabolism of proteins, Regulation of Insulin-like Growth Factor XBP1(S) activates chaperone genes
4	ACTB	Actin, cytoplasmic 1 OS = Homo sapiens GN = ACTB PE = 1 SV = 1	41.7	5.2	594.3	13	32.3	Actins are highly conserved proteins that are involved in various types of cell motility and are ubiquitously expressed in all eukaryotic cells.	Protein folding Signaling, signal transduction, Vesicle mediated transport, Clathrin-mediated endocytosis Developmental biology Cell-cell communication Disease oncogenic signaling
5					95.7	2	7.1	Required for the assembly and/or stability of the 40S ribosomal subunit. Required for the processing of the 20S rRNA-precursor to mature 18S rRNA in a late step of the maturation of 40S ribosomal subunits. Also functions as a cell surface receptor for laminin. Plays a role in cell adhesion to the basement membrane and in the consequent activation of signaling transduction pathways. May play a role in cell fate determination and tissue morphogenesis. Acts as a PPP1R16B-dependent substrate of PPP1CA	Viral mRNA translation Influenza viral RNA transcription and replication rRNA processing in the nucleous and cytosol
	RSSA	40S ribosomal protein SA OS = Homo sapiens GN = RPSA PE = 1 SV = 4	32.8	4.6					
6					101.9	2	7.1		
7	CH60	60 kDa heat shock protein, mitochondrial OS = Homo sapiens GN = HSPD1 PE = 1 SV = 2	61.0	5.6	322.9	7	12.4	Implicated in mitochondrial protein import and macromolecular assembly. May facilitate the correct folding of imported proteins. May also prevent misfolding and promote the refolding and proper assembly of unfolded polypeptides generated under stress	Gene expression Protein localization

								conditions in the mitochondrial matrix.	
	HNRPK	Heterogeneous nuclear ribonucleoprotein K OS = Homo sapiens GN = HNRNPK PE = 1 SV = 1	50.9	5.3	117.5	3	6.5	One of the major pre-mRNA-binding proteins. Binds tenaciously to poly(C) sequences. Likely to play a role in the nuclear metabolism of hnRNAs, particularly for pre-mRNAs that contain cytidine-rich sequences. Can also bind poly(C) single-stranded DNA. Plays an important role in p53/TP53 response to DNA damage, acting at the level of both transcription activation and repression. When sumoylated, acts as a transcriptional coactivator of p53/TP53, playing a role in p21/CDKN1A and 14-3-3 sigma/SFN induction (By similarity). As far as transcription repression is concerned, acts by interacting with long intergenic RNA p21 (lincRNA-p21), a non-coding RNA induced by p53/TP53. This interaction is necessary for the induction of apoptosis, but not cell cycle arrest	RNA metabolism<br>Disease infectious disease<br>Protein metabolism
8	HS90B	Heat shock protein HSP 90-beta OS = Homo sapiens GN = HSP90AB1 PE = 1 SV = 4	83.2	4.8	677.2	14	20.7	Molecular chaperone that promotes the maturation, structural maintenance and proper regulation of specific target proteins involved for instance in cell cycle control and signal transduction. Undergoes a functional cycle that is linked to its ATPase activity. This cycle probably induces conformational changes in the client proteins, thereby causing their activation. Interacts dynamically with various co-chaperones that modulate its substrate recognition, ATPase cycle and chaperone function.	Chaperone mediated autophagy<br>Signal transduction<br>Cellular response to external stimuli heat stress<br>Metabolism oxidation
	HS90A	Heat shock protein HSP 90-alpha OS = Homo sapiens GN =	84.6	4.8	598.8	15	22.1	Molecular chaperone that promotes the maturation, structural maintenance and proper regulation of	Cell cycle

		HSP90AA1 PE = 1 SV = 5						specific target proteins involved for instance in cell cycle control and signal transduction. Undergoes a functional cycle that is linked to its ATPase activity. This cycle probably induces conformational changes in the client proteins, thereby causing their activation. Interacts dynamically with various co-chaperones that modulate its substrate recognition, ATPase cycle and chaperone function. Binds bacterial lipopolysaccharide (LPS) et mediates LPS-induced inflammatory response, including TNF secretion by monocytes.	Chaperone mediated autophagy<br>Disease, signaling in cancer<br>Drug resistance<br>Signal transduction<br>Iinnate immune system<br>Metabolism of NO<br>Influenza infection<br>Gene expression, gene silencing<br>Interleukin 4 and 13 signalling<br>Organelle biogenesis and maintenance
9	ENPL	Endoplasmin OS = Homo sapiens GN = HSP90B1 PE = 1 SV = 1	92.4	4.6	535.9	14	15.7	Molecular chaperone that functions in the processing and transport of secreted proteins. When associated with CNPY3, required for proper folding of Toll-like receptors (By similarity). Functions in endoplasmic reticulum associated degradation (ERAD). Has ATPase activity.	Immune system<br>Metabolism of proteins<br>chaperon activate, regulation of insuli-like GF<br>Immune system<br>interleukin 4 and 13 signaling<br>Vesicle mediated transport
10	HNRPK	Heterogeneous nuclear ribonucleoprotein K OS = Homo sapiens GN = HNRNPK PE = 1 SV = 1	50.9	5.3	81.8	1	1.9	The same as for spot No. 7	Metabolism of RNA<br>Disease infectious<br>Metabolism of proteins
11	ALBU	Serum albumin OS = Homo sapiens GN = ALB PE = 1 SV = 2	69.3	5.9	212.3	4	7.1	Serum albumin, the main protein of plasma, has a good binding capacity for water, Ca^{2+} , Na^{+}, K^{+}, fatty acids, hormones, bilirubin and drugs. Its main function is the regulation of the colloidal osmotic pressure of blood. Major zinc transporter in plasma, typically binds about 80% of all plasma zinc.	Metabolism of proteins, regulation of insulin-like GF<br>Metabolism of porophyrins, bile acid and salt<br>Transport of small molecules

								Vesicle mediated transport Hemostasis, platelet activation, signaling and activation
12	RLA0	60S acidic ribosomal protein P0 OS = Homo sapiens GN = RPLP0 PE = 1 SV = 1	34.3	5.6	103.5	3	14.2	Ribosomal protein P0 is the functional equivalent of E.coli protein L10,	Gene expression, transcription Metabolism of proteins
13	LDHB	L-lactate dehydrogenase B chain OS = Homo sapiens GN = LDHB PE = 1 SV = 2	36.6	5.7	161.2	3	10.5	This protein is involved in step 1 of the subpathway that synthesizes (S)-lactate from pyruvate, m.in..	Metabolism, respiratory and electron transport
14	UCHL1	Ubiquitin carboxyl-terminal hydrolase isozyme L1 OS = Homo sapiens GN = UCHL1 PE = 1 SV = 2	24.8	5.2	58.8	1	4.5	Ubiquitin-protein hydrolase involved both in the processing of ubiquitin precursors and of ubiquitinated proteins. This enzyme is a thiol protease that recognizes and hydrolyzes a peptide bond at the C-terminal glycine of ubiquitin. Also binds to free monoubiquitin and may prevent its degradation in lysosomes. The homodimer may have ATP-independent ubiquitin ligase activity	Metabolism of proteins, deubiquitination

Table S3. Expected metabolic pathways assigned to results obtained on the base of transcriptome and proteome analyses. The frequency of the metabolic pathway representation is given in brackets.

Expected Metabolic Pathways		
**Metabolic Pathway Number**	**Transcriptome**	**Proteome**
1		Autophagy (3)
2		Cell–cell communication (1)
3		Cellular response (1)
4	Autophagy (1)	Developmental biology (3)
5	Cell—cell communication (1)	Disease (4)
6	Cellular response (1)	Gene expression (3)
7	Developmental biology (3)	Immune system (4)
8	Disease (3)	Metabolism (14)
9	Gene expression (7)	Organelle maintenance (3)
10	Immune system (6)	Protein localization (1)
11	Metabolism (11)	Signal transduction (3)
12	Organelle maintenance (1)	Transport of small molecules (1)
13	Protein localization (1)	Chaperon mediated processes (5)
14	Signal transduction (5)	Cell cycle regulation (2)
15	Transport of small molecules (2)	Cytoskeletal reorganization (1)
16		Drug resistance (1)
17	Extracellular matrix organization (1)	Haemostasis (1)
18	Chromatin organization (3)	Vesicle mediated transport (3)
19		Protein folding (1)
20		RNA processing (1)
21		Viral translocation (1)
